# A Novel Multi-Component Reaction to Imidazo[4,5-g]-quinazolines

**DOI:** 10.3390/molecules18055697

**Published:** 2013-05-16

**Authors:** Li Li, Qianqian Zhang, Bo Liu, Gang Liu

**Affiliations:** 1 Beijing Key Laboratory of Active Substances Discovery and Druggability Evaluation, Institute of Materia Medica, Chinese Academy of Medical Sciences & Peking Union Medical College, 1 Xian Nong Tan Street, Beijing 100050, China; E-Mails: zhangqianqian@imm.ac.cn (Q.Z.); liubo@imm.ac.cn (B.L.); 2 Tsinghua-Peking Center for Life Sciences and Department of Pharmacology and Pharmaceutical Sciences, School of Medicine, Tsinghua University, Haidian District, Beijing 100084, China

**Keywords:** multi-component reaction, heterocyclic compounds, imidazo[4,5-g]-quinazoline, structure determination, hetero Diels-Alder reaction

## Abstract

The trace impurities discovered after extended storage of a 5-amino- benzimidazole library were determined as imidazo[4,5-g]quinazoline derivatives by extensive spectroscopic data analysis. The formation of this highly aromatic heterocyclic ring involved a novel multi-component reaction, using which several novel compounds were prepared. Its mechanism was deduced as a cascade of chemical transformations, including the formation of a Schiff’s base, intramolecular hetero-Diels-Alder reaction, defluorination and dehydrogenation.

## 1. Introduction

**Scheme 1:**
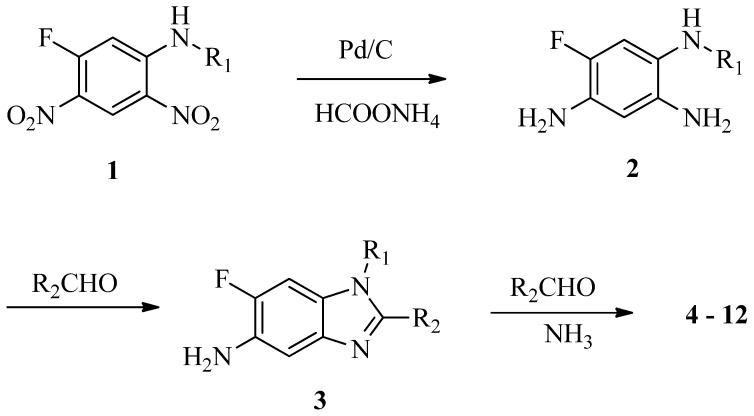
Chemical pathway to compounds **4**−**12**.

Benzimidazole has been regarded as a privileged sub-structure in fragment-based drug design and its derivatives have achieved great importance in pharmacology [1,2,3,4]. As a part of our program to prepare versatile chemical libraries from 1,5-difluoro-2,4-dinitrobenzene (DFDNB), we have disclosed a multistep parallel solution-phase synthesis of 5-aminobenzimidazole derivatives [5]. Then, a chemical library including 2,000 members was prepared. Most of the compounds showed high purity in the quality control by high-performance liquid chromatography/electrospray ionization mass spectroscopy (HPLC-ESIMS) method. However, it was found that trace impurities could often be detected in the wells containing 1-substituted-6-fluoro-2-(pyridin-4-yl)-1H-benz d]imidazol-5-amines (**3**, Scheme 1) after extended storage. All of them have positive mode MS peaks at 173 a.m.u. higher than the corresponding normal products **3**. This greatly stirred our interest in determining their chemical structures and establishing their origin. A systematic study including structure identification and chemical synthesis of these novel compounds was thus carried out and the results are presented here.

## 2. Results and Discussion

### 2.1. Structure Elucidation of the Trace Impurity

To obtain the trace impurity, the multi-step synthesis of 5-aminobenzimidazole derivatives was first performed on a larger scale than for the library preparation. 4-Pyridinylaldehyde and 3-isopropoxypropyl-1-amine were used in the model reaction. When the content of the M+173 impurity **4** was more than 1% by HPLC-ESIMS analysis, it was isolated by silica column chromatography. Then, its chemical structure was established by extensive spectroscopic data analysis.

Compound **4** was obtained as a yellow powder. Its molecular formula was determined as C_30_H_27_N_7_O from the HRESIMS ion at *m/z* 502.2353 [M+H]^+^ (calcd. 502.2349), which indicated 21 degrees of unsaturation. Since the normal product 6-fluoro-1-(3-isopropoxypropyl)-2-(pyridin-4-yl)-1*H*-benzo[*d*]imidazolyl-5-amine should have a molecular formula C_18_H_21_FN_4_O with 10 degrees of unsaturation, three nitrogen atoms and 11 degrees of unsaturation should have been introduced in the formation of compound **4**. It was noticed that 4-pyridinecarbaldehyde was added in twice the amount of reduction intermediate **2** and might still stay with compound **3**, even after adequate washing with aqueous sodium bicarbonate solution. Ammonia was also thought to be in the reaction mixture because of its release from residual ammonium formate. Thus, two more pyridinyl groups and a new aromatic ring containing an additional nitrogen atom were presumed to exist in the chemical structure of compound **4** compared with **3**.

**Figure 1:**
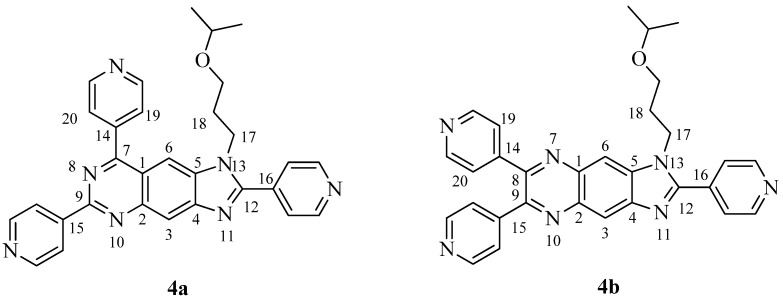
Two possible chemical structures of compound **4**.

The ^1^H-NMR spectrum of **4** confirmed the existence of an alkyl chain, three pyridinyl rings and a large conjugated aromatic ring. All signals in the high-field region could be attributed to the 3-isopropoxypropyl group. Six pairs of aromatic protons should be assigned to the three pyridinyl rings. It was obvious that both signals of H-3 and H-6 appeared as singlets, which should be split into a doublet because of heteronuclear coupling with the fluorine atom in compound **3** [5]. This indicated the absence of a fluorine atom in **4**, consistent with the HRESIMS results. The ^13^C-NMR and DEPT spectra of **4** showed 30 signals, including two primary carbons, three secondary carbons, 15 tertiary carbons and 10 quaternary carbons. These spectroscopic data suggested that **4** was a highly aromatic heterocyclic compound. Thus, two possible chemical structures **4a** and **4b** were proposed for **4**, which differed in the positions of one aromatic nitrogen atom and one pyridinyl ring (Figure 1).

The structure of **4** was further determined by comprehensive 2D NMR data analysis. In the NOESY spectrum, the correlation of H-6 with H-17 and H-18 indicated they were on the same side of the core aromatic ring. Meanwhile, NOESY correlation of H-6 with H-19 was observed, revealing that they were also on the same side and relatively close. In the optimized conformations of **4a** and **4b** using the MMFF94 molecular mechanical force field, the distances between H-6 and H-19 were 2.30 and 4.41 Å, respectively. This indicated that **4a** was the right chemical structure of compound **4**, which was further confirmed by gCOSY, gHSQC and HMBC spectra. Therefore, the chemical structure of **4** was established as 1-(3-isopropoxypropyl)-2,6,8-tri(pyridin-4-yl)-1*H*-imidazo[4,5-g]quinazoline.

### 2.2. Preparation of Imidazo[4,5-g]quinazoline Derivatives

**Figure 2:**
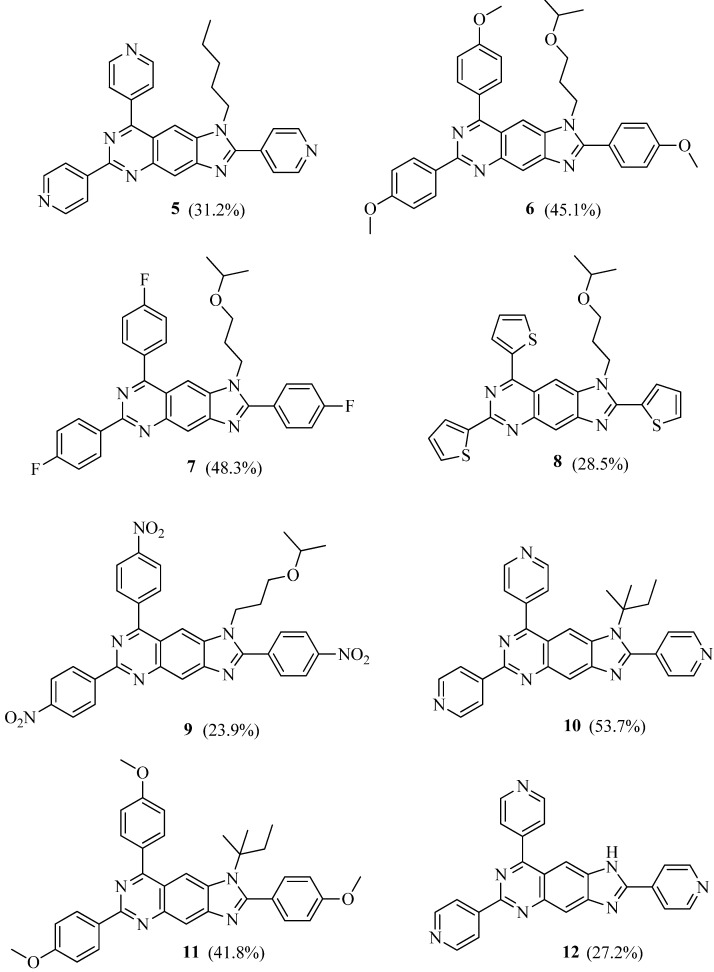
Chemical structures of compounds **5**–**12**.

Quinazolines represent a large family of compounds with significant pharmacological properties and other industrial uses [6,7,8]. Many methods for preparing quinazolines have been reported in the literature ([9,10], but to the best of our knowledge, there are no reports on the synthesis of 2,6,8-triaryl-1*H*-imidazo[4,5-g]quinazoline derivatives). Thus, the chemical transformation from 6-fluoro-1*H*-benzo[d]imidazol-5-amines to imidazo[4,5-g]quinazolines was investigated. Besides 4-pyridine carbaldehyde, three kinds of 4-substituted benzaldehyde and thiophene-2-carbaldehyde were used to give compounds **5**–**12** (Figure 2). 

Though no impurity was discovered in the wells of the counterpart in the 5-aminobenzimidazole library, all aromatic aldehydes could give the anticipated product after prolonged heating. It was found that the addition of ammonium hydroxide could promote the transformation, indicating that ammonia might participate in the reaction as supposed above. When aliphatic 2-ethylbutanal was used, no corresponding tricyclic product was detected. Meanwhile, it seemed that the substituents on the nitrogen atom have little effect on the reactivity of the intermediate **3**. 

The experimental procedure was similar to that of the preparation of 5-aminobenzimidazole library and performed in a sequential manner [5]. The reduction intermediate **2** was not purified and directly condensed with aldehyde to give condensation intermediate **3**, which was further reacted with additional aldehyde and ammonia. The total yield of the typical compound **4** might vary from 16.8% to 63.4% because of the instability of the key intermediate 2,4,5-benzenetriamines **2**.

### 2.3. Possible Mechanism of the Novel Multi-Component Reaction

**Scheme 2:**
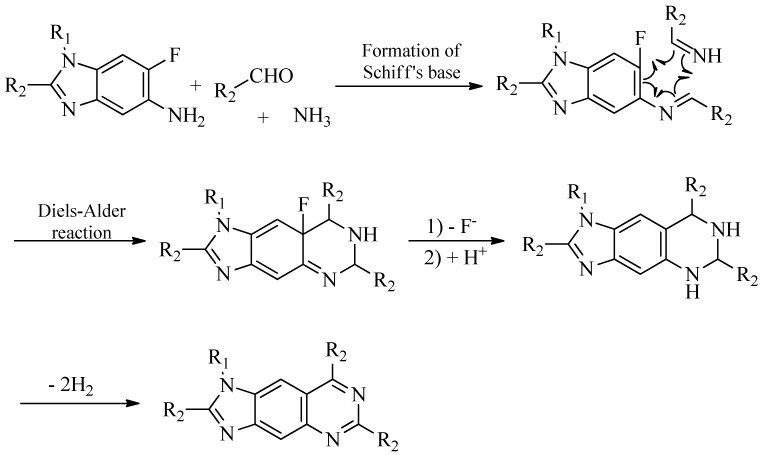
Plausible mechanism of the multi-component reaction.

Multi-component reactions have attracted considerable interest for their high synthetic efficiency [11,12,13]. This novel multi-component reaction might involve the formation of a Schiff’s base, intramolecular hetero Diels-Alder reaction, defluorination and dehydrogenation, as shown in Scheme 2. The loss of a fluorine atom might be a little difficult to explain, but all the supposed intermediates could be detected during the course of reaction. In the synthesis of **4** monitored by HPLC-ESIMS analysis, the main component at 2 h was a 5-amino-6-fluorobenzimidazole derivative with a positive mode MS peak at *m/z* 329.3. However, intermediates corresponding to positive mode MS peaks at 524.2, 506.2 and 504.2 appeared as the reaction progressed. In the end, a molecule corresponding to a positive mode MS peak at *m/z* 502.2 was the final product. Thus, the proposed mechanism was supported by the experimental evidence.

The hetero-Diels-Alder reaction has been regarded as one of the most convenient routes to N-heterocycles and has been used in the synthesis of many natural products [14,15,16,17]. It was also deduced as the key step in the present process, where the dienophile was the Schiff’s base formed between ammonia and the aldehydes.

## 3. Experimental

### 3.1. General

All chemical reagents were of reagent grade and used as purchased. Auto HPLC-ESIMS analysis was performed on a ThermoFinnigan, LCQ-Advantage mass spectrometer equipped with an Agilent 1100 HPLC system and a flow splitter. HPLC-HRESIMS were recorded on an Agilent 1100 series LC/MSD trap mass spectrometer (ESI-TOF). ^1^H-NMR and ^13^C-NMR spectra were recorded on a Mercury 300 or Inova 500 MHz NMR spectrometers equipped with an auto sampler. Infrared spectra were recorded using KBr pellets on a Nicolet Impact 400 spectrometer. Flash column chromatography was carried out on silica gel (200–300 mesh).

### 3.2. General Synthesis Procedure

To a solution of 0.5 mmol of the substituted dinitro compound **1** in tetrahydrofuran (8 mL) and ethanol (8 mL), HCOONH_4_ (800 mg) and 10% Pd-C (100 mg) were added under stirring. When the reaction mixture turned colorless, the catalyst and insoluble excess HCOONH_4_ were filtered off. The filtrate was added directly into a solution of aldehyde (1.5 mmol) and glacial acetic acid (1.2 mL) in tetrahydrofuran (4 mL). After stirring at room temperature overnight, the reaction mixture was heated to 90 °C for 36 h or longer. The reaction mixture was then evaporated to dryness, and the residue was further purified by silica gel column chromatography eluting with ethyl acetate/petroleum ether. For a typical product, *1-(3-isopropoxypropyl)-2,6,8-tri(pyridin-4-yl)-1H-imidazo[4,5-g]-quinazoline* (**4**), 159 mg of yellow powder was obtained (63.4% overall yield for three steps). M.p. > 300 °C; ^1^H-NMR (500 MHz, DMSO-*d_6_*): δ 0.79 (6H, d, *J* = 6.5 Hz), 1.88 (2H, m), 3.16 (2H, m), 3.20 (1H, m), 4.55 (2H, t, *J* = 7.5 Hz), 7.94 (2H, d, *J* = 5.5 Hz), 7.97 (2H, d, *J* = 5.5 Hz), 8.28 (1H, s), 8.46 (2H, d, *J* = 6.0 Hz), 8.56 (1H, s), 8.80 (2H, d, *J* = 5.5 Hz), 8.86 (2H, d, *J* = 5.5 Hz), 8.91 (2H, d, *J* = 5.5 Hz). ^13^C-NMR (125 MHz, DMSO-*d_6_*): δ 21.63 (2C), 29.37 (1C), 41.92 (1C), 63.42 (1C), 70.47 (1C), 106.90 (1C), 117.24 (1C), 117.91 (1C), 121.79 (2C), 123.53 (2C), 124.36 (2C), 136.80 (1C), 137.81 (1C), 144.70 (1C), 144.76 (1C), 146.40 (1C), 148.14 (1C), 150.18 (2C), 150.39 (2C), 150.45 (2C), 154.94 (1C), 158.06 (1C), 166.53 (1C). HRMS (ESI): *m/z* 502.2353 [M+H]^+^ (calcd 502.2349).

*1-Pentyl-2,6,8-tri(pyridin-4-yl)-1H-imidazo[4,5-g]quinazoline* (**5**): m.p. > 300 °C; yield: 31.2%. ^1^H-NMR (300 MHz, DMSO-*d_6_*): δ 0.72 (3H, t, *J* = 6.6 Hz), 1.13 (4H, m), 1.69 (2H, m), 4.46 (2H, t, *J* = 7.5 Hz), 7.90 (2H, d, *J* = 4.5 Hz), 7.97 (2H, d, *J* = 4.5 Hz), 8.28 (1H, s), 8.45 (2H, d, *J* = 4.5 Hz), 8.56 (1H, s), 8.80 (2H, d, *J* = 4.5 Hz), 8.87 (2H, d, *J* = 4.5 Hz), 8.91 (2H, d, *J* = 4.5 Hz). ^13^C-NMR (75 MHz, DMSO-*d_6_*): δ 13.58 (1C), 21.31 (1C), 27.98 (1C), 28.40 (1C), 44.33 (1C), 106.82 (1C), 117.16 (1C), 117.78 (1C), 121.68 (2C), 123.41 (2C), 124.35 (2C), 136.74 (1C), 137.50 (1C), 144.54 (1C), 144.62 (1C), 146.20 (1C), 147.94 (1C), 150.08 (2C), 150.33 (2C), 150.44 (2C), 154.77 (1C), 157.87 (1C), 166.30 (1C). HRMS (ESI): *m/z* 472.2249 [M+H]^+^ (calcd 472.2244).

*1-(3-Isopropoxypropyl)-2,6,8-tris(4-methoxyphenyl)-1H-imidazo[4,5-g]quinazoline* (**6**): m.p. > 300 °C; yield: 45.1%. ^1^H-NMR (300 MHz, CDCl_3_): δ 0.97 (6H, d, *J* = 6.3 Hz), 2.03 (2H, m), 3.28 (2H, m), 3.35 (1H, m), 3.90 (6H, s), 3.95 (3H, s), 4.46 (2H, t, *J* = 7.2 Hz), 7.04 (2H, d, *J* = 6.9 Hz), 7.08 (2H, d, *J* = 6.9 Hz), 7.15 (2H, d, *J* = 6.9 Hz), 7.84 (2H, d, *J* = 8.7 Hz), 7.94 (2H, d, *J* = 8.7 Hz), 8.10 (1H, s), 8.51 (1H, s), 8.69 (2H, d, *J* = 8.7 Hz). ^13^C-NMR (125 MHz, CDCl_3_): δ 22.11 (2C), 30.37 (1C), 42.51 (1C), 55.60 (1C), 55.70 (2C), 64.27 (1C), 71.89 (1C), 106.20 (1C), 114.02 (2C), 114.31 (2C), 114.62 (2C), 116.93 (2C), 118.27 (2C), 122.08 (1C), 130.38 (2C), 131.31 (2C), 131.49 (1C), 131.92 (2C), 136.95 (1C), 148.24 (1C), 158.08 (1C), 160.04 (1C), 161.27 (1C), 161.67 (1C), 161.81 (1C), 167.81 (1C). HRMS (ESI): *m/z* 589.2815 [M+H]^+^ (calcd 589.2809).

*2,6,8-Tris(4-fluorophenyl)-1-(3-isopropoxypropyl)-1H-imidazo[4,5-g]quinazoline* (**7**): m.p. > 300 °C; yield: 48.3%. ^1^H-NMR (300 MHz, DMSO-*d_6_*): δ 0.97 (6H, d, *J* = 6.0 Hz), 2.02 (2H, m), 3.29 (2H, m), 3.35 (1H, m), 4.45 (2H, t, *J* = 7.2 Hz), 7.21 (6H, m), 7.93 (4H, m), 8.06 (1H, s), 8.56 (1H, s), 8.72 (2H, m). ^13^C-NMR (100 MHz, DMSO-*d_6_*): δ 21.61 (2C), 29.31 (1C), 41.75 (1C), 63.43 (1C), 70.47 (1C), 106.75 (1C), 115.40 (1C), 115.60 (1C), 115.86 (1C), 115.90 (1C), 115.96 (1C), 116.12 (1C), 117.33 (1C), 125.95 (2C, d, *J* = 2 Hz), 130.22 (2C, d, *J* = 9 Hz), 132.33 (2C, d, *J* = 9 Hz), 134.26 (2C, d, *J* = 2 Hz), 134.31 (2C, d, *J* = 2 Hz), 137.09 (1C), 146.71 (1C), 148.02 (1C), 155.72 (1C), 159.15 (1C), 163.24 (1C, d, *J* = 246 Hz), 163.47 (1C, d, *J* = 248 Hz), 163.70 (1C, d, *J* = 246 Hz), 167.00 (1C). HRMS (ESI): *m/z* 553.2215 [M+H]^+^ (calcd 553.2211).

*1-(3-Isopropoxypropyl)-2,6,8-tri(thiophen-2-yl)-1H-imidazo[4,5-g]quinazoline* (**8**): m.p. > 300 °C; yield: 28.5%. ^1^H-NMR (300 MHz, CDCl_3_): δ 1.08 (6H, d, *J* = 6.0 Hz), 2.22 (2H, m), 3.46 (2H, m), 3.50 (1H, m), 4.68 (2H, t, *J* = 7.5 Hz), 7.21 (1H, d, *J* = 4.8 Hz), 7.25 (1H, d, *J* = 4.8 Hz), 7.32 (1H, d, *J* = 5.1 Hz), 7.51 (1H, d, *J* = 4.8 Hz), 7.65 (1H, d, *J* = 5.1 Hz), 7.69 (1H, d, *J* = 4.5 Hz), 7.93 (1H, d, *J* = 3.3 Hz), 8.00 (1H, d, *J* = 3.3 Hz), 8.25 (1H, s), 8.48 (2H, s). ^13^C-NMR (125 MHz, DMSO-*d_6_*): δ 22.10 (2C), 30.16 (1C), 41.48 (1C), 63.06 (1C), 70.13 (1C), 107.15 (1C), 119.25 (1C), 120.53 (1C), 125.16 (1C), 126.57 (1C), 127.95 (1C), 128.49 (1C), 129.05 (1C), 135.29 (1C), 136.91 (1C), 143.84 (1C), 143.62 (1C), 146.08(1C), 146.35 (1C), 146.82 (1C), 146.91 (1C), 149.05 (1C), 150.11 (1C), 155.04 (1C), 157.64 (1C), 167.42 (1C). HRMS (ESI): *m/z* 517.1190 [M+H]^+^ (calcd 517.1185).

*1-(3-Isopropoxypropyl)-2,6,8-tris(4-nitrophenyl)-1H-imidazo[4,5-g]quinazoline* (**9**): m.p. > 300 °C; yield: 23.9%. ^1^H-NMR (300 MHz, DMSO-*d_6_*): δ 0.77 (6H, d, *J* = 6.6 Hz), 1.86 (2H, m), 3.15 (2H, m), 3.22 (1H, m), 3.88 (6H, s), 3.92 (3H, s), 4.48 (2H, m), 7.35 (2H, d, *J* = 7.2 Hz), 7.49 (2H, d, *J* = 7.2 Hz), 8.01 (2H, d, *J* = 7.2 Hz), 8.26 (2H, d, *J* = 7.2 Hz), 8.46 (1H, s), 8.55 (2H, d, *J* = 7.2 Hz), 8.67 (1H, s), 8.90 (2H, d, *J* = 7.2 Hz). ^13^C-NMR (75 MHz, DMSO-*d_6_*): δ 23.04 (2C), 30.98 (1C), 42.19 (1C), 64.27 (1C), 75.21 (1C), 107.29 (1C), 116.38 (1C), 116.72 (1C), 117.19 (1C), 117.56 (1C), 119.12 (1C), 123.84 (2C), 124.08 (2C), 129.47 (2C), 130.47 (2C), 137.35 (2C), 137.69 (1C), 137.98 (2C), 142.18 (1C), 150.55 (1C), 161.01 (1C), 161.84 (1C), 162.61 (1C), 163.09 (1C), 164.16 (1C), 170.62 (1C). HRMS (ESI): *m/z* 634.2049 [M+H]^+^ (calcd 634.2045).

*1-(tert-Pentyl)-2,6,8-tri(pyridin-4-yl)-1H-imidazo[4,5-g]quinazoline* (**10**): m.p. > 300 °C; yield: 53.7%. ^1^H-NMR (300 MHz, DMSO-*d_6_*): δ 0.67 (3H, t, *J* = 7.2 Hz), 1.49 (6H, s), 2.05 (2H, m), 3.41 (6H, s), 3.44 (3H, s), 7.90 (2H, d, *J* = 4.5 Hz), 7.95 (2H, d, *J* = 4.5 Hz), 8.21 (1H, s), 8.43 (2H, d, *J* = 4.5 Hz), 8.50 (1H, s), 8.83 (2H, d, *J* = 4.5 Hz), 8.81 (2H, d, *J* = 4.5 Hz), 8.90 (2H, d, *J* = 4.5 Hz). ^13^C-NMR (75 MHz, DMSO-*d_6_*): δ 8.15 (1C), 29.22 (2C), 33.26 (1C), 61.15 (1C), 106.90 (1C), 117.19 (1C), 117.83 (1C), 121.28 (2C), 123.06 (2C), 124.13 (2C), 136.42 (1C), 137.39 (1C), 144.01 (1C), 144.48 (1C), 146.10 (1C), 147.69 (1C), 150.32 (2C), 150.43 (2C), 150.95 (2C), 154.70 (1C), 157.64 (1C), 166.15 (1C). HRMS (ESI): *m/z* 472.2247 [M+H]^+^ (calcd 472.2244).

*2,6,8-Tris(4-methoxyphenyl)-1-(tert-pentyl)-1H-imidazo[4,5-g]quinazoline* (**11**): m.p. > 300 °C; yield: 41.8%. ^1^H-NMR (300 MHz, CDCl_3_): δ 0.56 (3H, t, *J* = 7.5 Hz), 1.46 (6H, s), 2.02 (2H, m), 3.78 (6H, s), 3.80 (3H, s), 7.03 (2H, d, *J* = 7.2 Hz), 7.07 (2H, d, *J* = 7.2 Hz), 7.12 (2H, d, *J* = 7.2 Hz), 7.78 (2H, d, *J* = 7.2 Hz), 7.89 (2H, d, *J* = 8.4 Hz), 8.09 (1H, s), 8.50 (1H, s), 8.64 (2H, d, *J* = 8.4 Hz). ^13^C-NMR (125 MHz, CDCl_3_): δ 8.09 (1C), 29.14 (2C), 33.17 (1C), 55.14 (1C), 55.57 (2C), 61.19 (1C), 106.18 (1C), 114.05 (2C), 114.26 (2C), 114.65 (2C), 116.28 (2C), 118.01 (2C), 121.95 (1C), 130.16 (2C), 131.09 (2C), 131.32 (1C), 131.83 (2C), 136.90 (1C), 148.12 (1C), 158.00 (1C), 160.07 (1C), 161.05 (1C), 161.59 (1C), 161.72 (1C), 167.56 (1C). HRMS (ESI): *m/z* 559.2708 [M+H]^+^ (calcd 559.2704).

*2,6,8-Tri(pyridin-4-yl)-1H-imidazo[4,5-g]quinazoline* (**12**): m.p. > 300 °C; yield: 27.2%. ^1^H-NMR (300 MHz, DMSO-*d_6_*): δ 7.93 (2H, d, *J* = 4.8 Hz), 7.99 (2H, d, *J* = 4.8 Hz), 8.26 (1H, s), 8.49 (2H, d, *J* = 4.8 Hz), 8.54 (1H, s), 8.81 (2H, d, *J* = 4.8 Hz), 8.86 (2H, d, *J* = 4.8 Hz), 8.92 (2H, d, *J* = 4.8 Hz), 11.90 (1H, s). ^13^C-NMR (75 MHz, DMSO-*d_6_*): δ 106.78 (1C), 117.01 (1C), 117.69 (1C), 121.57 (2C), 123.28 (2C), 124.21 (2C), 136.56 (1C), 137.47 (1C), 144.29 (1C), 144.53 (1C), 146.16 (1C), 147.88 (1C), 150.26 (2C), 150.41 (2C), 150.49 (2C), 154.69 (1C), 157.76 (1C), 166.19 (1C). HRMS (ESI): *m/z* 402.1459 [M+H]^+^ (calcd 402.1462).

## 4. Conclusions

In conclusion, a novel multi-component reaction, which led to the formation of the unexpected trace impurities detected in a 5-aminobenzimidazole chemical library after extended storage, has been investigated. The possible reaction mechanism is thought to involve a cascade of chemical transformations, with the key step being a hetero Diels-Alder reaction. Its application to the preparation of other 6-halo-1*H*-benzo[d] imidazol-5-amines and *ortho*-haloanilines will be fully investigated in the future. 
